# 567. Performance of IgM and IgG antibodies against *Bartonella henselae* for diagnosis of Cat Scratch Disease in pediatric patients

**DOI:** 10.1093/ofid/ofad500.636

**Published:** 2023-11-27

**Authors:** Ana M Caratozzolo, Analía Toledano, María Laura Praino, Fausto Martín Ferolla, Silvina Neyro, Claudia Inés Cazes, Eduardo L López

**Affiliations:** Pediatric Infectious Diseases Program, Hospital de Niños "Dr. Ricardo Gutiérrez", Universidad de Buenos Aires, Buenos Aires, Ciudad Autonoma de Buenos Aires, Argentina; Pediatric Infectious Diseases Program, Hospital de Niños "Dr. Ricardo Gutiérrez", Universidad de Buenos Aires, Buenos Aires, Ciudad Autonoma de Buenos Aires, Argentina; Hospital General de Niños Dr. Ricardo Gutiérrez, Buenos Aires, Ciudad Autonoma de Buenos Aires, Argentina; Hospital General de Niños Dr. Ricardo Gutiérrez, Buenos Aires, Ciudad Autonoma de Buenos Aires, Argentina; Dirección de Control de Enfermedades Inmunoprevenibles, Ministry of Health,, Palermo, Ciudad Autonoma de Buenos Aires, Argentina; Hospital General de Niños Dr. Ricardo Gutiérrez, Buenos Aires, Ciudad Autonoma de Buenos Aires, Argentina; Pediatric Infectious Disease Program, Hospital de Niños Ricardo Gutiérrez, Universidad de Buenos Aires, Buenos Aires, Ciudad Autonoma de Buenos Aires, Argentina

## Abstract

**Background:**

*Bartonella henselae* infection causes Cat Scratch Disease (CSD). Lymphadenopathy is the most common symptom and a small percentage of patients have systemic impairment. CSD can mimic malignancy and manifest atypically, therefore reliable serological testing has clinical importance. CSD is diagnosed if two of the following criteria are met: 1) the presence of clinical symptoms consistent with CSD; 2) the existence of antibodies against *B. henselae* IgM and/or IgG (1:256) (Fig.1). We aim to assess the accuracy and diagnostic utility of IgM and IgG in pediatric patients with suspected CSD.

**Fig. 1:**
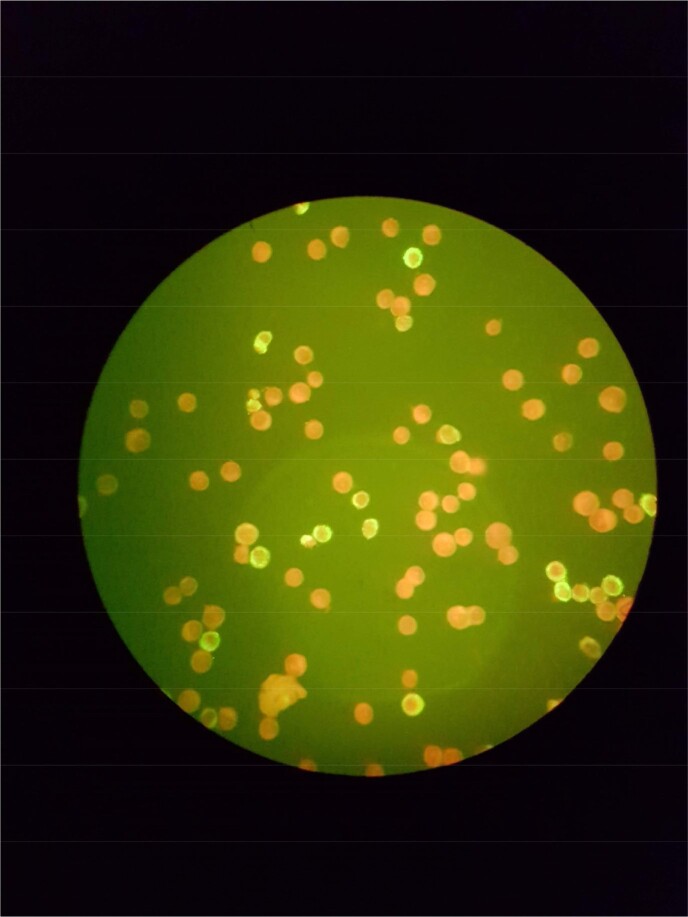
Immunofluorescence Assay Positive IgM against B. henselae

**Methods:**

A retrospective, observational and analytical study was carried out from April 2022 through April 2023. Medical and laboratory records of patients with follow-up in the Infectious Diseases Department of a Children's Hospital in Buenos Aires (Argentina) were evaluated.

**Results:**

We included 244 serum samples from patients with clinical suspicion of CSD using Immunofluorescent assay (IFA).

Median age: 84 (IQR 48-132) months, 137 were female, 95% (233/244) had cat interaction.

Twenty eight percent (70/244) were diagnosed with CSD based on serological evidence: 83,9% (58/70) had IgM +/IgG + (group 1); 10% (7/70), IgM-/IgG+ >1/64 (group 2) and 7,15% (5/70), IgM+/IgG – (group 3). The average time of symptom onset was 22,8 (6-90) days for group 1, 29,4 (11-90) days for group 2, and 11,4 (5-15) days for group 3.

Lymphadenopathy was found in 87,1% (61/70) with or without systemic involvement and 6 % (4/70) had systemic involvement without lymphadenopathy.

IgM had Sensitivity: 89%, Specificity: 97%, Positive Predictive Value (PPV): 92% and Negative Predictive Value (NPV) : 96%.

**Conclusion:**

Between 5-15 days after the onset of symptoms, IgM allowed us to diagnose 5 patients with CSD. The identification of IgM antibodies in CSD patients with systemic impairment has a great diagnostic performance. IgM remained positive after 90 days . We were able to diagnose CSD in 7 patients between 11 and 28 days following the onset of symptoms due to the presence of IgG 1:256 or higher. Combination of different methods (e.g., clinical and serological) and a correct time of sampling, greater than 5 days, should be considered for rapid and accurate CSD diagnosis.

**Disclosures:**

**All Authors**: No reported disclosures

